# Coverage with evidence development for medical devices in Europe: Can practice meet theory?

**DOI:** 10.1002/hec.4478

**Published:** 2022-02-26

**Authors:** Michael Drummond, Carlo Federici, Vivian Reckers‐Droog, Aleksandra Torbica, Carl Rudolf Blankart, Oriana Ciani, Zoltán Kaló, Sándor Kovács, Werner Brouwer

**Affiliations:** ^1^ Centre for Health Economics University of York York UK; ^2^ Centre for Research on Health and Social Care Management (CERGAS) Universitá Bocconi Milan Italy; ^3^ School of Engineering University of Warwick Coventry UK; ^4^ Erasmus School of Health Policy & Management Erasmus University Rotterdam The Netherlands; ^5^ Kompetenzzentrum für Public Management Universität Bern Bern Switzerland; ^6^ Swiss Institute for Translational and Entrepreneurial Medicine Bern Switzerland; ^7^ Syreon Research Institute Budapest Hungary; ^8^ Centre for Health Technology Assessment Semmelweis University Budapest Hungary; ^9^ Erasmus School of Economics Erasmus University Rotterdam Rotterdam The Netherlands

**Keywords:** cost‐effectiveness analysis, real‐world evidence, reimbursement

## Abstract

Health economists have written extensively on the design and implementation of coverage with evidence development (CED) schemes and have proposed theoretical frameworks based on cost‐effectiveness modeling and value of information analysis. CED may aid decision‐makers when there is uncertainty about the (cost‐)effectiveness of a new health technology at the time of reimbursement. Medical devices are potential candidates for CED schemes, as regulatory regimes do not usually require the same level of efficacy and safety data normally needed for pharmaceuticals. The purpose of this research is to assess whether the actual practice of CED for medical devices in Europe meets the theoretical principles proposed by health economists and whether theory and practice can be more closely aligned. Based on decision‐makers' perceptions of the challenges associated with CED schemes, plus examples from the schemes themselves, we discuss a series of proposals for assessing the desirability of schemes, their design, implementation, and evaluation. These proposals, while reflecting the practical challenges with developing CED programs, embody many of the principles suggested by economists and should support decision‐makers in dealing with uncertainty about the real‐world performance of devices.

## 1 INTRODUCTION

Coverage with evidence development (CED) is one form of performance‐based risk‐sharing agreements (PBRSA) that has been proposed to aid the entry of new health technologies into the healthcare system (Garrison et al., 2013). The distinctive feature of CED is that reimbursement of the new technology (drug, device, or procedure) is temporary, and conditional on further data being collected to reduce uncertainties concerning its clinical or cost‐effectiveness. CED is particularly relevant to medical devices because the evidentiary requirements for placing a device on the market are less demanding than for some other technologies, such as pharmaceuticals (Tarricone, Torbica, & Drummond, 2017). Thus, the available evidence for estimating the clinical and economic impact of medical devices is often less extensive and of lower quality.

In addition, collecting robust clinical evidence through randomized controlled trials (RCTs) may be unethical or infeasible. Furthermore, distinctive characteristics of medical devices, such as rapid incremental innovation and learning effects, present additional challenges for economic evaluation, and many of these factors are best assessed while the device is being used in regular clinical practice. Consequently, access to new medical devices may be delayed because of the absence of scientifically sound effectiveness evidence needed to respond to the expectations of policy‐makers deciding on reimbursement. In these situations, reimbursement on condition of gathering additional evidence may be an attractive option for all parties.

However, while CED offers a solution to some problems, it also presents several challenges of its own. These include determining when CED would be appropriate, designing and implementing the study, getting commitment from key stakeholders, funding the data collection and analysis, and generating results in a timely fashion (Drummond, 2015). In addition, there is a risk that patients may have access to a technology that is eventually found to be (cost‐)ineffective, and it may be difficult to restrict or withdraw the use of treatments at the end of the scheme, even if they do not live up to expectations (van der Wetering et al., 2017). The existence of these challenges makes decision‐makers less enthusiastic about CED than academic researchers or technology manufacturers (Dabbous et al., 2020; Schaffer et al., 2018).

Health economists and other scholars have written extensively on the design and implementation of CED schemes (Garrison et al., 2013) and have proposed theoretical frameworks based on cost‐effectiveness modeling and value of information analysis, but there may be challenges in applying these principles in practice to medical devices. Those responsible for funding health services research have argued that application of value of information (VOI) analysis would require significant resources, expertise, and planning, suggesting that the use of VOI in practice may remain limited (Fleurence & Selby, 2020). However, the use of VOI and other principles suggested in the health economics literature to date has not been studied in detail in the context of (practical applications of) CED.

The research reported in this paper is the third component of research into CED for medical devices conducted as part of the EU Horizon 2020 COMED (Pushing the Boundaries of Cost and Outcome Analysis of MEDical Technologies) project. The first component was a systematic review of 27 original articles and reviews on CED schemes for medical devices or health technologies more generally (Reckers‐Droog et al., 2020). The second component of the of the research used both literature searches and semi‐structured interviews with decision‐makers, to explore further the importance of the various challenges and to document the key characteristics of schemes undertaken, or currently in progress, in European countries between January 2014 and December 2019 (Federici et al., 2021). (Details of the previous research are given in Box 1.)

BOX 1 **Summary of previous components of the research**

1

**Table:** 

**Challenges with coverage with evidence development schemes for medical devices (MDs): A systematic review** (Reckers‐Droog et al., 2020) A systematic literature review was performed on six databases following PRISMA guidelines. Two independent reviewers assessed the eligibility of articles based on predefined criteria and extracted data from the included articles by using a pre‐defined extraction template. The results were synthesized in a qualitative review. The systematic search yielded 4293 articles of which 27 were eligible for inclusion. Twenty challenges associated with coverage with evidence development (CED) schemes for MDs were identified. Some of these challenges relate directly to the characteristics of MDs, and hence are specific to MDs. These challenges concern deciding on whether a CED scheme is required, understanding the relevant uncertainties and risks, identifying meaningful outcomes, defining an adequate duration for a scheme, and market entry of new technologies. Payers and manufacturers of MDs should address the identified challenges to improve a CED scheme's chance of success. This can be further improved by public sharing of information about the outcome of applied schemes and way in which stakeholders have addressed the challenges they faced when applying a CED scheme.
**Coverage with evidence development schemes for medical devices in Europe: characteristics and challenges** (Federici et al., 2021) Structured interviews were conducted with 25 decision‐makers from 22 European countries to explore the characteristics of existing CED programs for devices, and how decision‐makers perceived 13 pre‐identified challenges associated with initiating and operating CED schemes for devices. We also collected data on individual schemes that were either initiated or still ongoing in the last 5 years. Seven countries with CED programs for devices and 78 individual schemes were identified. The characteristics of CED programs varied across countries, including eligibility criteria, roles and responsibilities of stakeholders, funding arrangements, and type of decisions being contemplated at the outset of each scheme. A high variability in how decision‐makers perceived CED‐related challenges was observed possibly reflecting country‐specific arrangements and different experiences with CED. One general finding across all countries was that relatively little attention was paid to the evaluation of schemes, both during and at their completion.

This paper presents the third component of the research. Its main goal is to connect the theory behind CED schemes for medical devices with the practice in Europe, which has not been done previously. In doing so, the study uses a targeted review of the health economics literature on the theoretical framework underlying CED as well as the data obtained in the first two components of research. The study had the following objectives: (i) to assess the extent to which the theoretical principles proposed by health economists are followed in current practice of CED for medical devices in Europe; (ii) to gain an understanding of why practice may not (always be) aligned with theory; and (iii) to formulate recommendations on how practice and theory can be more closely aligned.

## 2 THE HEALTH ECONOMICS LITERATURE ON CED

### 2.1 Methodological considerations in designing CED schemes

The main methodological contribution by health economists to CED has arguably been in the use of VOI analysis for making reimbursement decisions. The logic behind VOI analysis is that the cost‐effectiveness of new technologies is uncertain, introducing the possibility of error into decisions concerning their adoption or use (decision uncertainty). Acquiring the necessary information through research could reduce uncertainty in the evidence base and the associated cost of making the wrong decision. However, generating scientific evidence is often costly and time‐consuming. VOI analysis provides a formal assessment of the value of research, based on the extent to which the new information improves the expected payoff associated with the decision by reducing uncertainty (Fenwick et al., 2020). This can then be compared with the cost of conducting the research.

VOI can be used to prioritize research, by estimating the value of acquiring perfect information about all aspects of the decision (i.e., the Expected Value of Perfect Information), or by estimating the value of perfect information about a specific (group of) parameter(s) in the decision (i.e., the Expected Value of Partial Perfect Information, EVPPI). It can also inform research design, by estimating the expected value associated with a given sample size and a particular design, which results in a reduction of decision uncertainty (i.e., the Expected Value of Sampling Information, EVSI; Fenwick et al., 2020).

VOI analysis can be extended to simultaneously evaluate the payoffs of alternative reimbursement and research design decisions and to estimate how different configurations impact population net‐benefits over time (McKenna & Claxton, 2011). Mirroring financial option pricing theory and its application to real‐world decisions, this approach was also defined as real‐options analysis (ROA). In ROA, VOI is used as an explicit analysis framework to estimate the payoffs of an immediate reimbursement decision against delaying the decision until new evidence has been generated (Fornaro et al., 2021; Grutters et al., 2011). In addition, VOI can be used iteratively over the life cycle of a technology and can be reassessed when an element of the decision changes, such as publication of new research, the emergence of a new comparator technology, or changes in the relative prices of technologies. These approaches fit very well with CED and the dynamic characteristics of medical devices (Grimm et al., 2017).

Rothery et al. (2017) have developed a formal approach for considering choices in research and technology adoption in the context of medical devices, based on VOI analysis, which can be used in situations where the assessment of the device has included the development of a cost‐effectiveness model. They point out that there are four main coverage decisions possible (approve, reject, only in research, and only with research), the last two of which could be the basis for a CED scheme. They show that the choice between these options depends on several factors, including the likely cost‐effectiveness of the device based on current evidence, the level of irrecoverable costs should use of the device need to be restricted or withdrawn (e.g., capital costs in equipment and facilities), the value of additional evidence, the incentives for the manufacturer, and other parties to conduct research, the likelihood of changes in the price of the device or its comparators, and the value to patients (and more broadly to society) of early access.

The practical challenges of implementing VOI analysis have also been acknowledged in this literature. Fenwick et al. (2020), for example, note that “Although VOI analyses are being increasingly published in academic journals, uptake in real‐world decision‐making remains limited. This is partially due to perceptions that a VOI analysis is complex to perform, difficult to interpret, requires substantial computational time and does not reflect key relevant uncertainties.”

Recent methodological developments have eased the computational burden of VOI by introducing regression‐based approaches to estimate EVPPI and EVSI. However, these methods still require that a full probabilistic cost‐effectiveness model is developed (Heath et al., 2017; Rothery et al., 2020), which is not always feasible for medical devices. Fenwick et al. (2020) note that in such situations a “rapid VOI” or a minimal modeling approach may be required. For example, Claxton et al. (2015) have shown how a simple extension of standard meta‐analysis of the clinical evidence can provide quantitative estimates of the potential health benefits of further research, which can help inform research prioritization and efforts to change clinical practice.

### 2.2 Economic aspects of CED in practice

There have been several literature reviews on PBRSAs (including CED), mostly focusing on pharmaceuticals but sometimes discussing other health technologies, including medical devices. (See Garrison et al. (2013) and Stafinski et al. (2010) for good examples.) In addition, Gerkens et al. (2017) identify several key points based on Belgian and international experiences of managed entry agreements (MEA) for pharmaceuticals, including PBRSAs. Also, again focusing on pharmaceuticals, a recent report for the OECD by Wenzl and Chapman (2020) identified five elements of good practice: (i) defining a strategy to guide use of PBRSAs for pharmaceuticals; (ii) ensuring they are used only where the benefit of additional evidence outweighs the cost of negotiating and executing the agreement; (iii) clearly identifying uncertainties in each reimbursement decision and design agreements to ensure that data sources and research designs are appropriate to address the uncertainties; (iv) implementing a governance framework that ensures transparency of process, and allows payers to act upon the additional evidence, including exiting from the agreement and potential withdrawal of temporary coverage; and (v) ensuring a minimum level of transparency of content, limiting confidentiality to those parts of the agreement that may be commercially sensitive (in particular, prices).

Economists have also discussed whether there are any distinctive characteristics of medical devices that might challenge or complicate the conduct of CED schemes. Drummond et al. (2009) note that product modifications frequently occur and may affect the performance of the device; as physicians use the device there may be a learning effect (or “curve”) and consequent improvements in effectiveness of the procedure (Varabyoova et al., 2017); prices of devices are often more dynamic than those of pharmaceuticals and are more likely to change over time, thereby affecting estimates of relative costs and cost‐effectiveness. These factors are often ignored in economic evaluations (Enzing et al., 2021; Tarricone, Torbica, & Drummond, 2017) but suggest that CED schemes may require re‐analysis over time in order to inform the conditional reimbursement decisions to which they pertain.

Finally, in several settings, including Europe and the United States, devices can enter the market by claiming “substantial equivalence” with a similar existing device, thereby reducing the need to produce additional evidence on effectiveness. This may lead to uncertainty about the true level of equivalence of various devices and puts the main burden for evidence generation on the manufacturer of the first device of that type entering the market. Therefore, new devices claiming “substantial equivalence” may be candidates for CED schemes and could be added to an ongoing scheme if one is operated. Claims of “equivalence” are likely to be less common under more recent EU guidance endorsed by the Medical Device Coordination Group established by Article 103 of Regulation (EU) 2017/745 (European Commission, 2020), but if a device enters the market that is like one in an existing CED scheme, consideration should be given to adding it to the scheme.

## 3 CED FOR MEDICAL DEVICES IN EUROPE: THEORY VERSUS PRACTICE

**FIGURE 1:**
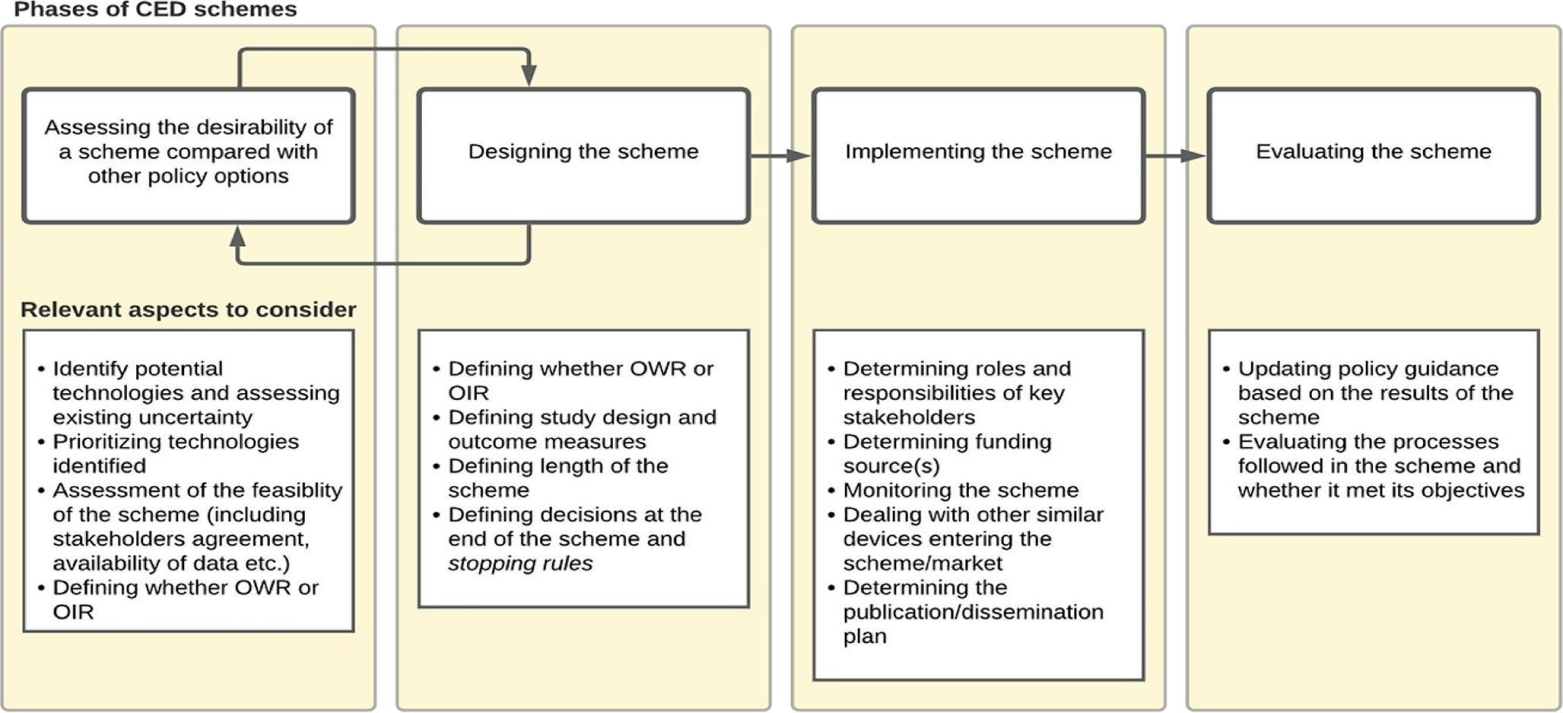
Phases of coverage with evidence development schemes for medical devices

To assess to what extent the theoretical principles proposed by health economists are met in practice, we examined the various European programs for CED of medical devices in more detail, in terms of the four phases discussed by Garrison et al. (2013). These are assessing the desirability of a CED scheme; designing a scheme; implementing a scheme; and evaluating a scheme (see Figure 1). Here we use this framework to develop proposals for the design, implementation, and use of CED schemes, in order to make these more consistent with the principles outlined in the health economics literature.

The first phase of a CED scheme relates to the way candidate devices for CED schemes are identified and the assessment on the value of a CED scheme as opposed to other available policy options (e.g., financial agreements, unconditional reimbursement, or rejection). The design phase concerns decisions on the specific features of the scheme such as the study design (e.g., registry‐based studies vs. randomized controlled studies) and the choice of outcome measures. These features may in turn affect the desirability of a scheme by altering its feasibility, the costs of data collection, and the expected returns in terms of reduced uncertainty. This correlation has been represented in the diagram by bi‐directional arrows between these two phases. The implementation phase concerns the different ways schemes are operated and how roles and responsibilities are distributed among the stakeholders involved (e.g., the funder or purchaser of health care, health technology assessment (HTA) agencies, manufacturers, or providers). Lastly, the evaluation phase mainly relates to two different aspects. The first concerns the updating of policy guidance about the device under assessment when the study reports its results (e.g., continued reimbursement, restricting, or delisting of the device), while the second concerns the evaluation of the processes that took place and whether the scheme met the objectives. The evaluation may be used to identify and resolve potential challenges or inefficiencies associated with the CED program for devices in place and to inform potential revisions as well as future schemes.

### 3.1 Assessing the desirability of CED schemes

The most common way of identifying potential candidate devices for CED schemes in Europe is through the reimbursement process, where a decision would be made on whether to include the device on a “positive list” for reimbursement, or guidance issued on its use in the healthcare system (Federici et al., 2021). Depending on the setting and type of device, this process may involve an assessment of device performance, safety, effectiveness, and/or cost‐effectiveness. For example, in France, the request to conduct a post‐registration study (PRS) can be issued for any technology for which relevant evidence gaps have been identified during the initial request by the manufacturer for registration in the list of reimbursable products and procedures (LPPR). A request for a PBRSA is explicitly made by the Medical Device and Health Technology Evaluation Committee (CNEDiMTS) and, if accepted by the Ministry of Health, renewal of the registration in the LPPR (after about 3 years) is made conditional to the provision of new evidence by the applicant.

Health economists argue that in assessing the desirability of a CED scheme, the various alternative policy options to establishing a scheme need to be considered, including (unconditionally) approving reimbursement for the device, refusing reimbursement, restricting use of the device to specific patient groups, or a price negotiation with the manufacturer (Walker et al., 2012). However, evidence of a formal assessment comparing all the (opportunity) costs and benefits of the different research and adoption policies was generally missing in most European countries with CED schemes for devices, although it is possible that these issues may have been considered informally.

Health economists also argue that CED should be considered only in situations where there is decision uncertainty about the safety, clinical, or cost‐effectiveness of the technology that could be reduced though further data collection (Drummond, 2015). A CED scheme should not be used merely as a mechanism for delaying a decision that could be taken immediately. Neither should price negotiation be used as a substitute for further data collection when substantial uncertainties in clinical effectiveness or safety remain. Price reductions can reduce the uncertainty and level of financial risk for the payer, but do not avoid the possibility of patients receiving treatment involving ineffective, or potentially harmful, devices.

If the assessment carried out as part of the reimbursement process included the development of an economic model and an assessment of the likely cost‐effectiveness of the device, it might be possible to be quite specific about whether a CED scheme would be useful. For example, writing in the context of the UK National Health Service (NHS), where the National Institute for Health and Care Excellence (NICE) undertakes assessments of new health technologies, Grimm et al. (2016) provide a checklist of questions to establish the potential need for a MEA if information is available on the likely cost‐effectiveness of the technology and the nature of the residual uncertainty (see Table 1).

**TABLE 1:** Five key questions in establishing the potential need for a PBRSA in a technology appraisal

Q1) Which intervention do we expect to be most cost‐effective given proposed prices and current evidence?
Q2) How uncertain are we?
Q3) How useful would it be to eliminate uncertainty?
Q4) Given current evidence and proposed prices, what is the strategy‐specific risk to the NHS?
Q5) How much would the NHS expect to gain by eliminating the risks associated with both uncertainty and the strategy?

Abbreviations: NHS, National Health Service; PBRSA, performance‐based risk‐sharing agreements.

*Source*: Grimm et al. (2016).

Even if a full economic model is not required, it is still essential that, prior to initiating a CED scheme, a detailed analysis is made of the important residual uncertainties and whether they can be resolved through CED. For example, in an analysis of CED dossiers for drugs in the Netherlands, Pouwels et al. (2019) highlighted that uncertainties could exist in effectiveness, safety, and impacts on quality of life and resource use. They then characterized the uncertainties as being related to *unavailability of evidence*, *indirectness of evidence* (e.g., lack of head‐to‐head clinical studies or evidence only from a different patient population or sub‐group), and *imprecision of evidence*. Characterizing the uncertainties in this way may help decision‐makers determine which residual uncertainties are most important for the relevant decision problem and whether a CED scheme could resolve them.

In contrast, an approach used in some European countries is to consider requests for devices to be included in a CED scheme made by key stakeholders, such as clinical groups, hospitals, or manufacturers. Often these requests will need to be prioritized before more detailed consideration of whether the residual uncertainties could be resolved by a scheme is possible. Criteria for priority setting could relate to the unmet need and disease severity of patients, size of the clinical impact (in improved effectiveness or reduced risk), or the likely size of the economic impact if the device were to be adopted for regular clinical use. Ideally, such requests would have the support of multiple stakeholders and meet pre‐defined standards.

**FIGURE 2:**
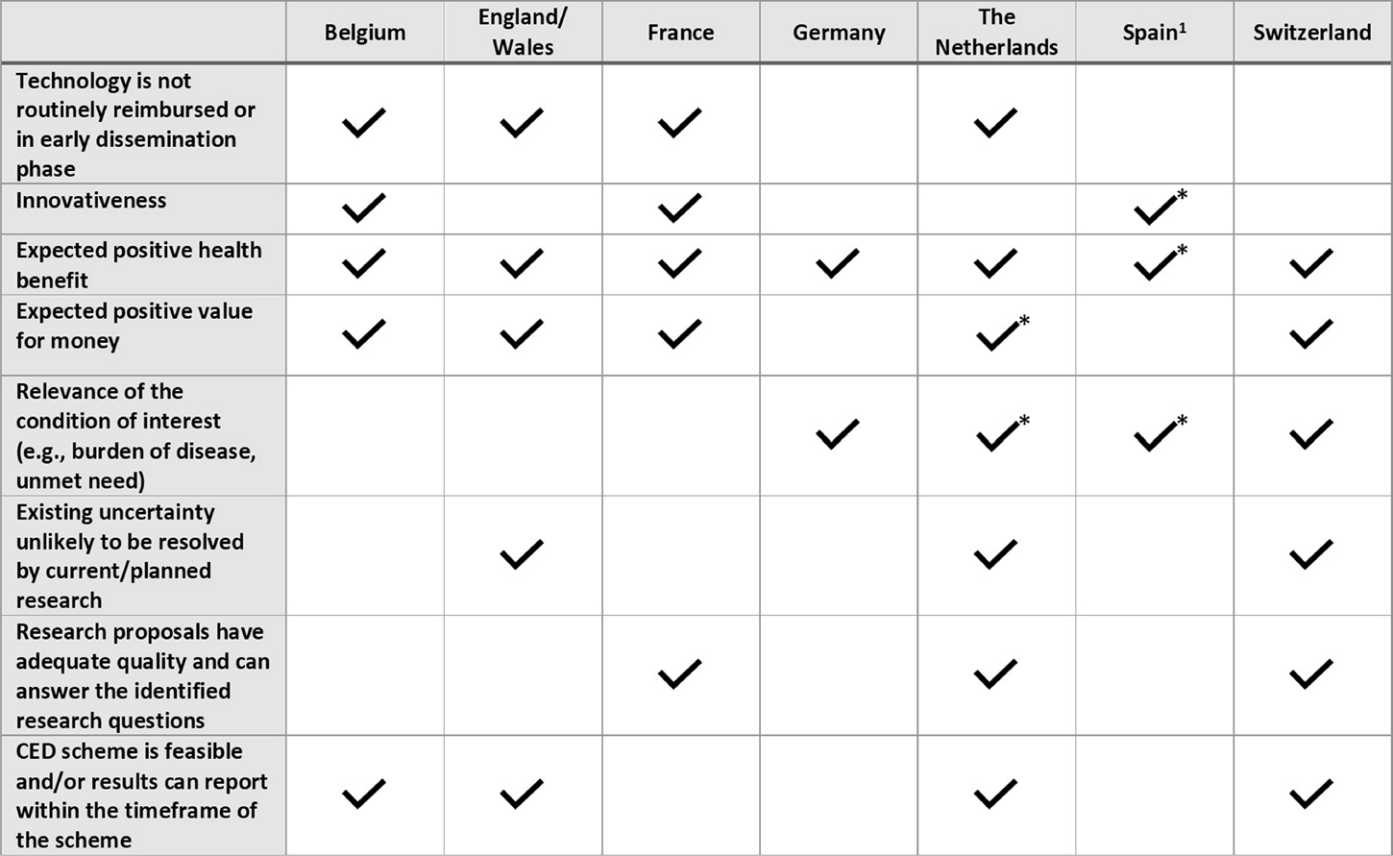
Criteria for the selection and prioritization of schemes in seven countries*. *Criterion used only for prioritization, not for eligibility; ^1^In Spain, proposal for schemes are put forward by the individual regions, using their own eligibility criteria. Subsequently, proposals are prioritized within the inter‐regional council using an explicit quantitative tool that includes a list of 15 weighted criteria across four domains: population/end users (e.g., disease burden, frequency of use); technology (e.g., innovativeness, different expectations of use); Safety/adverse effects (e.g., safety issues, undetected adverse effects); organization/costs and other implications (e.g., learning curve, financial impact, organizational or structural impact)

The criteria for the prioritization and selection of schemes in the seven European countries currently operating CED programs are described in Figure 2. In some countries (i.e., England, France, Germany, and Belgium), a deliberative approach is taken based on pre‐specified criteria, while in others (i.e., Spain, the Netherlands, and Switzerland), quantitative tools or checklists are used to select and prioritize the schemes. It needs to be recognized that such selection and prioritization is especially important since the resources (financially and otherwise) available for CED schemes are typically limited, especially when they are publicly financed. Therefore, technologies need to be prioritized for use in CED schemes in such a way as to maximize the expected ultimate returns of the research investments. Again, VOI provides an explicit framework that allows explicit comparisons of the costs and benefits of specific study designs across different research proposals and technologies.

Health economists argue that another important aspect of assessing the desirability of a CED scheme is whether the device should be approved only in research (OIR) or only with research (OWR; Carlson et al., 2010). In the case of OIR, the device is only reimbursed for a subset of patients enrolled in a clinical study. Under OWR, the device is reimbursed for all eligible patients, but on the condition that further data are collected. In practice, this choice is likely to be made based on the type of residual uncertainty. For example, if there are doubts about clinical effectiveness or safety, OIR is more likely to be preferred. Therefore, the choice between OIR and OWR also needs to be considered in the study design phase. (See Section 3.2). Specific institutional arrangements and available policy options also play a role here. Claxton et al. (2016) have developed a comprehensive algorithm to guide the choice between OIR and OWR plus, as mentioned in Section 2.1, Rothery et al. (2017) have developed a formal approach for considering these policy choices in the context of medical devices, based on VOI analysis, which can be used in situations where assessment of the device included the development of a cost‐effectiveness model.

However, of the seven European countries with CED programs for medical devices in place, only in England, the Netherlands, and Belgium have cost‐effectiveness models been developed to inform reimbursement decisions for medical devices. In addition, national programs for CED tended to focus exclusively on either OIR or OWR schemes rather than considering them as alternative policy choices. One of the key determinants of the choice between OIR and OWR was the minimum level of evidence that was required for a device to be admittable to the scheme.

For example, in the French post‐registration studies, and the Spanish post‐introduction observation studies, despite residual uncertainty, technologies admitted to the schemes were expected to provide an added value compared to existing alternatives, and the authorities were (already) considering reimbursing the technology for a large proportion of the patient population. In these schemes, CED were always configured as OWR and decisions at the end of the scheme never resulted in a withdrawal of the technology, but rather in refinements to the conditions of their use (e.g., based on further long‐term data on specific subpopulations). However, an assessment of the consequences of providing broader access to a technology as opposed to more restrictive designs such as OIR was never formally addressed. In other countries such as Germany (for inpatient technologies) and Switzerland, CED schemes were initiated when the technology was already adopted in clinical practice, so OIR was generally considered unfeasible or unethical.

### 3.2 Designing CED schemes

Once it has been determined that a CED scheme would be desirable, thought needs to be given to the design of the scheme. As well as being a key component of whether to have a scheme, the choice between OIR and OWR is also one important aspect of study design since it influences several methodological choices, most importantly the choice of the study population. In addition, detailed consideration of other aspects of study design could lead to a reconsideration of the desirability of a CED scheme, for example, if the research required to reduce the key uncertainties raises several practical challenges that first have to be resolved.

One of the main features of the study design is whether an RCT is required and feasible, or whether an observational study will (have to) suffice. Similarly, the choice between OIR and OWR, in the European CED programs, the preferred study design tends to be constant within each program and is often a reflection of the scope of schemes and the types of uncertainty they aim to address. Some of the European CED programs state a preference for RCTs, citing that this represents the “highest level of evidence.” In these programs, insistence on RCT evidence is justified by the fact that the most relevant uncertainty in the schemes concerns the relative treatment effect, and the performance of the new device compared with the current standard of care. Indeed, in this case it has been argued by some health economists that observational evidence from CED schemes is insufficient (Grieve et al., 2016). However, RCTs are difficult to conduct for some categories of devices due to ethical or practical considerations. For example, patient recruitment can be difficult to secure after market entry if the device is reimbursed and widely available. Therefore, conducting an RCT is probably easier when following the “only in research” approach, although one study found that, for an implantable device, the Conformité Européenne (CE) mark to allow market entry was awarded before the RCTs were completed, suggesting that the OIR approach may sometimes be difficult to sustain in practice (Tarricone et al., 2016).

Other CED programs are more focused on addressing uncertainty related to the real‐world performance of the device. For example, schemes may be initiated to explore the durability of the clinical effect, the device's performance in different patient sub‐groups, the impact of the learning effect on performance, or the rate of uptake and conditions of use, including potential off label uses of the device in regular clinical practice. For example, in 2013 NHS England initiated a so‐called “Commissioning through Evaluation” scheme for percutaneous mitral valve leaflet repair for primary degenerative mitral regurgitation in adults (Mitraclip) and purposely set up a registry to collect data on outcomes and safety and inform later decisions. The primary outcomes of the study were implantation success rates, in‐hospital major and minor complications, as well as post‐discharge complications and mitral regurgitation over time.

In these cases, schemes mainly rely on evidence from observational data, such as that from registries, to answer the relevant research questions. However, in all cases where an observational study is being conducted, it is important to ensure that data are collected on relevant covariates (e.g., risk level of patients) to facilitate adequate adjustments for confounders. Other research conducted in the COMED project has explored the different methods for analyzing observational data on medical devices (Pongiglione et al., 2022) and its use by HTA bodies in Europe (Klein et al., 2022).

Another important issue is the choice of outcome measure(s) on which to base the scheme. Ideally this would be a (final) outcome of relevance to patients that is easily measurable and clearly attributable to the performance of the device under consideration. For instance, a CED scheme could be mandated to clarify the link between an initially observed effect on a surrogate endpoint and the final patient‐relevant outcome of interest, thus establishing the validity of a putative surrogate. In fact, almost all CED schemes on devices that have been operated in the last 5 years in Europe, collected data on final endpoints, including meaningful clinical endpoints, health‐related quality of life, and/or other patient reported outcomes. A major focus was also placed on real‐world device performance and safety, such as the incidence of adverse events, medium and long‐term device durability, device failures or malfunction rates, and the number of required corrective surgeries following the index procedure.

Lastly, some European schemes also explored real‐world use, such as the number of procedures and patients' characteristics, or adherence rates, and/or the impact of the device on resource use and costs or cost‐effectiveness. However, on occasions it may be necessary to still measure a surrogate outcome in the scheme, in which case it is important to ensure that it has been appropriately validated and contributes to meeting the scheme's objective. Other research undertaken as part of the COMED project has included a review of the methodological guidance on the use of surrogate endpoints issued by HTA agencies in Europe, Australia, and Canada (Grigore et al., 2020) and a mapping of approaches to handle surrogate validation in HTA reports across eight HTA bodies internationally (Ciani et al., 2021). A methodological framework and policy tool for the evaluation of health technologies that rely on evidence from surrogate endpoints has also been developed (Ciani et al., 2022).

It is important to discuss the intended duration of the scheme. In the European countries, schemes for medical devices usually last between 2 and 5 years (Federici et al., 2021). Health economists have argued that the length of the scheme should be primarily driven by the evidence requirements, with the qualification that long‐term schemes (e.g., longer than 3 years) risk losing the interest of decision‐makers (Drummond, 2015). In some countries, the length tends to be the same across all schemes, whereas in others length is decided on a case‐by‐case basis, based on the requirements of the data collection and the study protocol. For example, in England, commissioning through evaluation (CtE) schemes tend to have a fixed 2‐year duration, whereas in the French Forfait Innovation package the duration of the scheme, including the periods for data collection, data analysis, and reassessment by the health authority, is specified for each technology in the national decree approving the temporary reimbursement.

Setting the same prescribed length for all schemes may be administratively convenient and facilitate scheduling of the negotiations with the manufacturer at the end on the scheme, but it may not be the best choice, as optimal length is affected by the key areas of uncertainty to be addressed in the scheme and the time needed to generate adequate data. As mentioned, ROA and VOI analyses may be used to formally assess the optimal length of the scheme alongside other design features of the schemes. In addition, beyond the initially planned length of the scheme, it is important to establish an appropriate monitoring of the scheme, which should also include a “stopping rule,” or “exit rule” based on an interim assessment of the progress of the scheme and/or the data generated. For example, mechanisms should be put in place to ensure that recruitment in the study is proceeding, the data is of sufficient quality and that all the parties engaged in the scheme are performing as expected. This aspect is particularly relevant especially when the responsibility for data collection and analysis relies mostly on the manufacturers or healthcare providers (see Section 3.3).

Finally, health economists argue that consideration should be given at the outset to the possible actions on the conclusion of the scheme (Drummond, 2015). For example, what would the scheme need to demonstrate to be considered a success? Would the device need to achieve a pre‐specified level of clinical or cost‐effectiveness, or provide a minimum required level of evidence, for reimbursement to be made permanent? Will the predefined level of achievement need to be reached, in particular pre‐specified patient sub‐groups? The feasibility and implementation of potential final decisions, including withdrawal require attention in this context as well.

However, pre‐specifying criteria for a future decision can be challenging. For example, if a scheme is planned to operate for 2–3 years, it is possible that other changes in clinical practice, including the development of other new treatments and devices, may impact on the cost‐effectiveness of the device. Few examples of pre‐specifying decision‐making criteria were identified in our study of current European practice. However, until 2019, CED schemes in the Netherlands were regulated under the conditional admission (VT) program. Under this program, the scheme requirements were part of a binding contract (covenant) between the relevant parties (researchers, care providers, professional groups, patient organizations, and manufacturers). The covenant was drawn up under the supervision of the Dutch National Health Care Institute (ZIN). Specifically, the agreement regulated the essential requirements that had to be met before the intervention could be conditionally reimbursed from the basic benefits package of the mandatory health insurance, the obligations of the parties during the implementation of the CED scheme, and the possible scenarios at the end of the scheme, including the case that the device did not permanently enter into the basic benefits package.

### 3.3 Implementing CED schemes

CED schemes often involve a range of stakeholders and this can create challenges in terms of communication and clarification of responsibilities. Therefore, it is important from the outset to determine who is responsible for the governance of the scheme and for mediating any disputes between stakeholders during the operation of the scheme. This may include elements like the development of the study protocol (based on the study design, discussed above), but also the recruitment of patients, the collection and analysis of data, and monitoring the general progress of the scheme. In part, the distribution of roles also depends on the party responsible for the funding of the scheme. If government or other public funding is available, it is less likely that key tasks will be sub‐contracted to the manufacturer. Typically, funding the scheme relates mainly to the data collection and analysis, since under most of the arrangements the device itself will be reimbursed for the patients participating in the scheme. The provision of adequate funding for data collection, to the participating clinical centers or individual physicians, may be important for the success of the scheme since the inadequacy of data collection is one of the most frequently reported problems by policy‐makers. It is important to establish an adequate infrastructure for data collection (e.g., electronic medical records) that can be implemented in all the participating clinical centers.

In some cases, the scheme applicant (e.g., a manufacturer, clinical group, or provider) has the responsibility for data collection and analysis. In other cases, for example, in England, these tasks may be sub‐contracted to an independent academic research center to preserve neutrality. There may be a trade‐off between the provision of public funding for schemes and the number of schemes that can be conducted. In France (in the PRS program), where funding for data collection and analysis is the responsibility of the manufacturer, CED schemes involved approximately 15% of all devices assessed for inclusion in the LPPR. On the contrary in countries such as England or Germany, where CED schemes are publicly funded and centrally managed, the overall number of schemes were more limited, 5 in England and 10 in Germany over the 5‐year period studied. However, where schemes are left to the responsibility of scheme applicants, it is important to discuss the burden of data collection with the relevant stakeholders in the scheme to ensure feasibility and to conduct continuous follow‐up to check the quality and validity of the data being collected. Some countries have considered funding schemes jointly between the public and private sectors (i.e., manufacturers), which may be one way of ensuring that both parties have a stake in generating good quality data.

The involvement of an independent academic research center may increase the chances of the results of the scheme being published. Transparency is not often a central requirement in CED schemes, but publication of the protocol, or posting it on an open science website, would be helpful to decision‐makers in other settings, including late technology adopters in, for example, Central and Eastern European countries (Daubner‐Bendes et al., 2021; Kovács et al., 2022). In a working paper produced for the OECD, Wenzl and Chapman (2020) argue that as many features of schemes as possible should be in the public domain, apart from confidential items such as the details of any financial settlement made following the scheme (e.g., on the price of the device). Features of schemes that could be made public are the study design and methodology, the new evidence generated by the scheme, and any policy recommendations that were made following the scheme.

Especially in the case of devices, the scheme may need to be adapted to take account of the distinctive characteristics of devices, such as product modifications, the learning effect and similar devices entering the market. In the case of product modifications and the learning effect, the most important aspect would be to log the time these occur, the level of changes, and the characteristics of the product user and organizational context. This can then be taken into account when the data are analyzed.

Policies also need to be developed to deal with similar products entering the market or further generations of the same product during a CED scheme. Some health economists have suggested that, if the scheme is being run by the healthcare system rather than an individual manufacturer, these devices could enter the scheme, thereby increasing the number of patient‐years of use during the scheme and to investigate whether similar devices are indeed equivalent to one another (Drummond et al., 2016). For example, in France, the manufacturer of a new version of a cardiac monitor, the REVEAL LINQ, that was conditionally approved in 2015, was required to provide data to the same post‐registration study of the previous version of the device (REVEAL XT), after the CNEDiMTS considered these two versions as having the same expected treatment effect (HAS, 2015, 2018). In some cases, the collection of new data may be provided in the setting of a broader national registry for a class of devices treating the same or similar indications. For example, since 2007, the CNEDiMTS has set up the EPIIC registry (Etude Post‐inscription des systems d’Implants Cochléaires et du tronc cérébral), with the aim of collecting data on the benefit of all registered cochlear implants for adults and children. Any new manufacturer of a cochlear implant submitting a demand for registration in the LPPT is required to provide data to the EPIIC registry.

However, other options are possible, including initiating a separate scheme for the new device. On occasions, it may be tempting for the payer to accept a price cut from the new device manufacturer, as has happened in Australia with pharmaceuticals (Wlodarczyk et al., 2011), but this would be inappropriate if the new device exhibited the same clinical uncertainties as the device already in a CED scheme. However, in our survey “Dealing with other similar devices entering the market” was not ranked among the most important issues (Federici et al., 2021). This suggests that the challenges posed by a new device entering the market during a scheme have not often been faced in Europe to date, or that these issues have not been widely discussed by decision‐makers.

### 3.4 Evaluating CED schemes

The evaluation of schemes consists of two elements. First, it can be useful, for the design and implementation of future schemes, to review how the data were collected, whether data collection was adequately monitored, and whether the final dataset met expectations in terms of being timely, complete, and of good quality. In addition, any practical problems arising during the conduct of the scheme, and the reasons for any delay in completion of the scheme should be noted.

The second important factor in the evaluation of CED schemes is whether any possible decisions to be taken at the conclusion of the scheme had been pre‐specified and whether the scheme has provided a basis for informing them. For example, will the reimbursement of the device be made permanent? If so, will this be for all patient groups, or just a subset of patients? Based on the demonstrated cost‐effectiveness of the device, does the current price represent good value for money? If not, what mechanisms exist to negotiate or enforce lower prices? How will future payment for the device be realized, for example, through a revision of a hospital diagnosis‐related group (DRG) payment? If the device is found not to be clinically or cost‐effective, what actions need to be taken to withdraw the device?

Health economics have argued that decision‐making and, especially, implementing the decision at the end of a scheme can be difficult. van der Wetering et al. (2017) point out that removing a health technology from the reimbursement list can be challenging, suggesting that removal of a device is likely to be found more difficult in the context of OWR than OIR schemes. All implementation decisions are likely to be more complex for medical devices than for pharmaceuticals. Many devices are used in a hospital setting and are funded as part of a bundled payment for the procedure in which the device is used (e.g., a DRG payment). These payments are reviewed periodically to account for inflation, but less frequently to allow for changes in practice, such as the adoption of a new procedure involving a device. Sorenson et al. (2015) argue that more should be done through these payments to incentivize hospitals to adopt new, cost‐effective, technologies and to discontinue those that are not. However, this is generally not the case, although in some cases ‘incentive payments’ are made to hospitals pending an update of the DRG tariff (Sorenson et al., 2013).

In addition, other research shows that the adoption of new devices is dependent on physicians' preferences (Hatz et al., 2017), so changing hospital payments may not necessarily change practice, unless physician fees are also related to use of device. Therefore, other initiatives may be required, such as developing clinical practice guidelines and applying peer pressure. In some cases, the HTA agency or other body overseeing a CED scheme may have some influence over these initiatives, but our research showed that often they did not. Therefore, ideally these bodies would be stakeholders in the scheme, as one key action at the end of a scheme would be to involve those organizations responsible for reviewing payment mechanisms or clinical practice guidelines.

Of the European schemes that ended between 2014 and 2019 and for which the results led to a final reimbursement decision, most resulted in the unrestricted and unconditional reimbursement of the device. An example of restricted reimbursement is intra‐arterial thrombolysis/thrombectomy for patients with acute cerebral infarction in the Netherlands. This treatment was conditionally reimbursed between 2013 and 2016. Based on the results of the scheme, the ZIN concluded that the treatment met the statutory criterion “Established Medical Science and Medical Practice” and, therefore, should in principle be reimbursed. However, the intervention was considered “highly complex,” and hence it was decided that the treatment would initially only be provided in selected specialized hospitals that met the required quality standards and have a “demonstrable research infrastructure” (ZIN, 2016). Moreover, these hospitals needed to be prepared to participate in research to further improve patient selection. An example of withdrawal from reimbursement are Renal Denervation procedures in the Netherlands. This procedure was also conditionally reimbursed between 2013 and 2016. Based on the results of this scheme, ZIN concluded that the collected evidence failed to demonstrate that the procedure met the statutory criterion “Established Medical Science and Medical Practice” and it was, subsequently, withdrawn from reimbursement in 2017.

An overall summary of the extent to which current practice deviates from health economists' recommendations is given in Table 2.

**TABLE 2:** Are health economists' recommendations being followed in practice?

Recommendations	Current practice
Assessing the desirability of CED schemes	
Determine the need for a scheme based on an HTA including an economic evaluation.	Partially followed
	Only few countries conduct an economic evaluation to inform reimbursement decisions for medical devices.
Use VOI and ROA approaches to inform on the desirability, prioritization, and design of schemes.	Not followed
	VOI/ROA never used in any of the countries with CED programs for devices.
Compare the costs and consequences of CED schemes with other, alternative policy options.	Not followed
	When deciding on a scheme, a formal, explicit assessment of the costs and consequences of all policy options was generally missing.
Only use CED when uncertainty can be reduced through further data collection.	Partially followed
	All countries use explicit criteria for the selection of schemes. In some countries the criteria include the possibility of uncertainty being resolved by the scheme
Design of a scheme	
The type of CED (e.g., OIR and OWR), and the study design (e.g., experimental vs. observational) should be informed by explicit assessments on appropriateness, costs, and consequences of each option.	Not followed
	The type of CED scheme and study design tended to be constant across the different national programmes, and not informed by an explicit evaluation
The outcomes measured in a CED should be final, relevant outcomes attributable to the device.	Mainly followed
	Almost all the schemes operated in Europe collected data on final endpoints, including meaningful clinical endpoints, health‐related quality of life and other patient‐reported outcomes
The length of the scheme should be primarily driven by the evidence requirements.	Partially followed
	In some countries the length tended to be the same across all schemes, whereas in others length was decided on a case‐by‐case basis. Few countries considered feasibility of data collection within the duration of the scheme.
Monitoring mechanisms, as well as stopping rules, should exist to ensure that schemes are proceeding as planned.	Partially followed
	Reports on progress with data collection and data quality are often envisioned, but stopping rules/exit rules are almost never clearly defined.
The criteria to inform policy actions at the outset of the scheme should be pre‐specified at the beginning of the scheme.	Not followed
	In practice, pre‐specifying decisions at the beginning of a scheme can be challenging. Only one example of an attempt to do this was identified.
Implementing CED schemes	
Clearly identify the key responsibilities of various parties in providing funding, developing the study protocol, collecting and analyzing data.Make the details of the scheme (e.g., uncertainties to be resolved, study design) publicly available.	Partially followed
	There is substantial variability in the type and amount of information available across the different countries. Few countries published the detail of the schemes at the outset. Some countries reported the results of the evidence generated through the schemes in appraisal reports.
Anticipate possible adjustments of CED schemes, to deal with similar products entering the market, or product modifications	Not followed
	In practice countries did not explicitly anticipate changes in CED schemes to deal with product modifications or similar products entering the market. Only one example of this was identified.
Evaluating schemes	
Assess whether the scheme achieved its aims.	Not followed/Not determined
	There was no information in the public domain to show that these assessments were made. However, the assessments may have been made privately
Make appropriate decisions on reimbursement, coverage or price of the device based on the results of the scheme	Partially followed/Not determined
	There were some examples of reimbursement restrictions made as a result of schemes. However, following most schemes the device was given unrestricted and unconditional reimbursement and it was hard to determine whether this was justified or not.

Abbreviations: CED, coverage with evidence development; HTA, health technology assessment; OIR, only in research; OWR, only with research; ROA, real‐options analysis; VOI, value of information.

## 4 DISCUSSION; ALIGNING PRACTICE WITH THEORY

It is clear from the analysis above that the practice of CED for medical devices in Europe deviates from the principles outlined in the health economics literature. However, can theory and practice be more closely aligned? We make six recommendations, as outlined in Table 3.

**TABLE 3:** Recommendations for aligning theory and practice in CED for medical devices

1) Define the purpose of the CED scheme in terms of the uncertainty to be resolved
2) Apply VOI where feasible, or at least VOI principles
3) Reflect the nature of the uncertainty in the study design
4) Balance scientific and practical considerations when determining the length of CED schemes
5) Define decisions to be taken at the end of the CED scheme as early as possible
6) Provide solid reasons when deviating from common CED principles

Abbreviations: CED, coverage with evidence development; VOI, value of information.

First, in assessing the desirability of schemes, it should be re‐affirmed that the purpose of CED schemes is to reduce the uncertainty surrounding the effectiveness or cost‐effectiveness of the new technology and that the nature of this uncertainty should be characterized. In those programs where a CED scheme is recommended following the results of a health technology assessment this may already be the case. However, it may not always be the case when schemes are requested by the manufacturer, hospitals, or clinical groups, so should be conducted as an additional requirement before initiating a scheme.

Second, although it may be unrealistic to expect a formal VOI analysis to be conducted in most cases, because of lack of expertise, the time and effort required or the lack of a cost‐effectiveness model, VOI principles should be applied informally, by considering the nature of the decision uncertainty about the cost‐effectiveness of the device, how useful it would be to reduce or eliminate this uncertainty, the cost of conducting the additional research and its likely success, and the costs of reversing the reimbursement decision in the future should that prove necessary.

Third, the study design for CED schemes should reflect the nature of the uncertainty surrounding the effectiveness or cost‐effectiveness of the device and practicalities of conducting the research. In cases where uncertainty relates to the use or effectiveness of the device in regular clinical practice (including the learning effect), the durability of clinical effect, or long‐term safety, an observational study would normally suffice. On the other hand, if the uncertainty relates to the relative clinical effect of the new device compared with current care, the potential biases of an observational study and the potential for correcting for bias through statistical analysis should be weighed against the practicalities of conducting an RCT. This assessment should also consider whether providing broader access to a technology would compromise the possibility of generating valuable evidence in the future.

Fourth, the length of the CED should be determined by a balance of scientific and practical considerations. On the one hand, if the allotted time is insufficient for recruiting enough patients or to observe changes in the outcome(s) of interest, the research may be inconclusive and the scheme may fail to meet its aim. On the other hand, if the time required to conduct the study is likely to make the results redundant, owing to changes in the availability or costs of other devices or treatment options, it may not be worthwhile undertaking.

Fifth, thought should be given to the decisions that will be taken at the end of the scheme (e.g., relating to the reimbursement, coverage, or price of the device), as well as the mechanism for implementing these decisions in the health care system. Since it is likely that the range of policy options will change over time, these considerations should be reviewed periodically during the operation of the scheme.

Finally, more consideration should be given to why practice in CED so often deviates from the principles outlined in the health economics literature. In commenting on the lack of adoption of VOI analyses by research funding bodies, Fleurence and Selby (2020) point to the computational burden of conducting analyses on a range of competing research projects. In the context of CED, it may be that decision‐makers do not only view schemes as being important for gathering the necessary information to make an optimal resource allocation decision, but also as part of a broader negotiation with manufacturers concerning the pricing and reimbursement of the new health technology. In that sense, one could argue that while the rationale and health economic background for having a CED may relate to determining what the optimal decision would be, more attention could be given in future work to the political, behavioral, and institutional aspects of reimbursement and pricing decisions, and the incentives regarding whether and how decisions can be informed and implemented. Both elements appear to be crucial in practice.

## 5 CONCLUSIONS

Current practice in CED for medical devices varies across countries in Europe and deviates from the proposals outlined in the health‐economic literature. While it is unlikely that theory and practice will (ever) be completely aligned, we have made several recommendations which, while reflecting the challenges associated with CED, embody many of the principles suggested by health economists and should contribute to reducing uncertainty about the real‐world performance of medical devices, thereby supporting decision‐makers in the development and conduct of CED schemes in the future.

## CONFLICT OF INTEREST

The authors have no conflicts of interests with respect to this research.

## Data Availability

The data that support the findings of this study are available from the corresponding author upon reasonable request.
